# Role of Ethnicity and Sex in Hypertension-Mediated Organ Damage in a Dual-Ethnic Cohort of Individuals With Hypertension

**DOI:** 10.1161/HYPERTENSIONAHA.125.25032

**Published:** 2025-10-20

**Authors:** Anna Hernandez-Rubio, Ryan John McNally, Núria Pedrós Barnils, Bushra Farukh, Phil J. Chowienczyk, J. Kennedy Cruickshank, Luca Faconti

**Affiliations:** School of Cardiovascular and Metabolic Medicine & Sciences, King’s College London British Heart Foundation Centre, United Kingdom (A.H.-R., B.F., P.J.C., J.K.C., L.F.).; Germans Trias i Pujol Research Institute, Badalona, Barcelona, Spain (A.H.-R.).; King’s Health Partners, Centre for Translational Medicine, King’s College London, United Kingdom (R.J.M.).; Institute for Public Health and Nursing Research, University of Bremen, Germany (N.P.B.).

**Keywords:** aldosterone, carotid-femoral pulse wave velocity, ethnicity, hypertension, kidney

## Abstract

**BACKGROUND:**

Ethnic and sex disparities in hypertension-mediated organ damage (HMOD) have been described. However, the intersection between ethnicity and sex in this context has not been extensively investigated. Moreover, whether activation of the renin-angiotensin-aldosterone system and aldosterone in particular could play a role is still unknown. Here, we aimed to investigate the intersection of sex and ethnicity with vascular and renal-HMOD, and its association with aldosterone.

**METHODS:**

Individuals with primary hypertension and self-defined ethnicity Black/White were included, and assessed for anthropometry, biochemistry, and vascular (carotid-femoral pulse wave velocity [cf-PWV]) and renal-HMOD (microalbuminuria/decreased filtration rate). Multivariate logistic, linear regression, and inverse probability weighting analyses were performed to study associations with cf-PWV/kidney damage, using the less affected group as reference. Associations of aldosterone were examined using adjusted-Pearson correlations.

**RESULTS:**

Overall, 654 individuals (age, 44 years) divided into 4 intersectional categories (Black women, Black men, White women, and White men) were included. Multivariate and inverse probability weighting analyses showed higher cf-PWV in Black women (β=1.25 m/s [95% CI, 0.77–1.73]) compared with White men and higher odds of kidney damage in Black individuals (Black men odds ratio, 4.16 [95% CI, 1.48–11.96] and Black women odds ratio, 3.83 [95% CI, 1.40–1.48]), compared with White women. Aldosterone was associated with kidney damage regardless of ethnicity and sex (odds ratio, 1.02 [95% CI, 1.01–1.03]), and showed a modest correlation with cf-PWV only in Black individuals (ρ=0.20, *P*=0.001).

**CONCLUSIONS:**

There are sex and ethnic differences in vascular and renal-HMOD, with Black women having increased cf-PWV and Black individuals having higher odds of kidney damage. Whether aldosterone could play an independent role in ethnicity-related HMOD differences would require further investigation.

**NOVELTY AND RELEVANCE**

**What Is New?**

This study introduces the novel integration of the intersection of sex and ethnicity to explore subgroup effects on vascular and kidney damage in hypertension. Using a well-phenotyped dual-ethnic cohort of individuals with hypertension living in the United Kingdom, the study employs fully adjusted multivariate models that are further included in an inverse probability weighting analyses to try to minimize confounding and get closer to causality assessment. Furthermore, potential associations with aldosterone are evaluated, addressing an important gap in knowledge.

**What Is Relevant?**

The identification of individuals at higher risk of hypertension-mediated organ damage is relevant to guide personalized approaches, enhancing intervention strategies based on their risks. In this regard, the findings of this study highlight that Black individuals living in the United Kingdom have an increased risk of kidney damage compared with White women, and Black women show higher levels of arterial stiffness compared with White men. Although aldosterone was independently associated with kidney damage in the overall population, and correlated with arterial stiffness in Black individuals, its role in explaining ethnic differences in hypertension-mediated organ damage remains uncertain and warrants further investigation.

**Clinical/Pathophysiological Implications?**

The study underscores the importance of considering ethnicity, sex, and aldosterone in the assessment of hypertension-mediated organ damage. A better understanding of the interaction of ethnicity, sex, and activation of the renin-angiotensin system could contribute to personalizing treatment for hypertension and tailored prevention strategies.

Hypertension is the major modifiable risk factor for cardiovascular morbidity and mortality.^1,2^ Ethnic disparities in the prevalence, severity, and rate of control of hypertension are well documented, especially among African-origin individuals living in the Northern Hemisphere.^2–5^ Hypertension in Black individuals is also associated with a higher rate of cardiovascular complications.^6^ Aldosterone has been identified as a key contributor to the development of hypertension and hypertension-mediated organ damage (HMOD),^7^ due to its role in promoting oxidative stress, endothelial dysfunction, inflammation, and fibrosis.^8^ Its involvement in cardiac HMOD has been well documented, particularly in subjects with a low renin hypertension phenotype.^9,10^ However, the extent to which aldosterone influences HMOD in renal and vascular tissues, and whether these effects vary by ethnicity and sex, remains a subject of ongoing investigation.

Similarly, sex differences in hypertension and cardiovascular disease have been described, with women presenting increased cardiovascular risk after menopause,^11^ but the interaction between sex and ethnicity is not well established.^12,13^

Here, we aimed to investigate the interaction between sex and ethnicity on HMOD in a dual-ethnic cohort of individuals with hypertension. We hypothesized that there are sex-specific ethnic differences in vascular and renal-HMOD, and these could be associated with differences in serum aldosterone concentration. To study the interaction between sex and ethnicity, we introduced intersectionality in the analyses. Intersectionality is an analytical framework rooted in the premise that human experience is jointly shaped by multiple factors (eg, sex, ethnicity), which can interact to affect health-related outcomes. Thus, quantifying intersectionality can help understand subgroup’s effects^14^ on the study of HMOD.

## Methods

### Data Availability

The data that support the findings of this study are available from the corresponding author on reasonable request.

### Study Population

A cross-sectional study of adult individuals (>18 years) with an established diagnosis of primary hypertension attending the hypertension outpatients’ clinic at Guy’s and St Thomas’ Hospital NHS Foundation Trust in London, England, United Kingdom, was performed following the Strengthening the Reporting of Observational Studies in Epidemiology guidelines (Supplemental Material). In the United Kingdom, individuals with hypertension are routinely referred from primary care for the exclusion of secondary causes of hypertension and treatment initiation in specific circumstances, especially if diagnosed before the age of 40 years.^15^ The study population was recruited from an area of south-east London characterized by a high proportion of people of non-White background.^16^

Individuals with hypertension were categorized based on their self-defined ethnicity in line with current routine practice in the United Kingdom, as Black and White. Individuals were recorded as Black if their self-described ethnicity from their clinical records was either Black-African, Black-Caribbean, Black-British, or Black. Individuals were included as White if their self-described ethnicity was White-British, White-Irish, White-European, or White others. Individuals who identified their ethnicity as mixed ethnicity or other ethnicities apart from the 2 described (Black and White) were not included in the study. Sex assigned at birth was recorded from all participants and is referred to as sex throughout the article. Intersectional categories considering sex and ethnicity resulted in the following 4 groups: White women, White men, Black women, and Black men.

Exclusion criteria (in addition to other ethnic backgrounds) included pregnancy, secondary hypertension, patients with heart failure, moderate or severe valvular disease, and those with sustained nonsinus arrhythmias. The study was approved by the local Research Ethics Committee in the UK, and written informed consent was obtained from all patients (approval number: IRAS 208017). The methods used in this study complied with the ethical standards for medical research and the principles of good clinical practice established in the Declaration of Helsinki.

### Variables and Definitions

Patients underwent an interview to collect data regarding cardiovascular risk factors, medical comorbidity, and history of previous cardiovascular disease. Anthropometric data (height, weight, body mass index, blood pressure, and heart rate measurements), carotid-femoral pulse wave velocity (cf-PWV), blood tests (including renal function, sodium, potassium, lipid profile, HbA1c [glycated hemoglobin], aldosterone, and renin), and urine (for the evaluation of the albumin to creatinine ratio), were collected without treatment washout. Hypertension was diagnosed based on the evidence from the medical record and/or evidence of daytime ambulatory blood pressure readings (or home blood pressure averaged over 7 days) of >135 mm Hg systolic or >85 mm Hg diastolic, according to current NICE (National Institute for Health and Care Excellence) guidelines.^15^

Main outcomes of the study were kidney damage and arterial stiffness, which were defined as follows. Renal function was assessed by the estimated glomerular filtration rate (eGFR) formula, calculated with the Chronic Kidney Disease Epidemiology Collaboration equation.^17^ Kidney damage was defined following the 2024 KDIGO (Kidney Disease: Improving Global Outcomes) guidelines^18^ as the presence of microalbuminuria (albumin to creatinine ratio in spot urine 30–300 mg/g or 3–30 mg/mmol), macroalbuminuria (albumin-creatinine ratio in spot urine >300 mg/g or >30 mg/mmol), and eGFR <60 mL/min.

Cf-PWV was used as a marker of arterial stiffness. ECG‐referenced carotid and femoral tonometry recordings were obtained using the SphygmoCor device.^19^ Measurements were made by a single operator, and the average of 2 consecutive measurements was used for the analysis.

### Statistical Analysis

Continuous variables were detailed using mean and SD or as median [interquartile range]. Categorical variables were detailed using relative frequencies. One-way ANOVA test was performed to assess differences in means of the quantitative variables among the 4 intersectional groups, whereas the χ^2^ test was used for differences in proportions of the categorical variables.

To address the potential correlation of aldosterone in HMOD ethnic and sex differences, we used an adjusted partial Pearson coefficient to assess its correlation with both eGFR and cf-PWV by ethnicity and sex, adjusting for the 2 main confounders (age and mean arterial blood pressure).

Linear regression was used to study the relationship between the 4 intersectional groups and cf-PWV. The baseline group was defined as the one with lower mean PWV and multivariate linear regression analysis was performed taking into account the following confounding variables for its known association with PWV from previous knowledge and its association with cf-PWV in the univariate analysis: age, mean blood pressure, heart rate, body mass index, creatinine, aldosterone and renin, as continuous variables; and smoking status, treatment for hypertension and diabetes, as binary variables.^20^ The *F* test was used to assess the overall significance of the regression model. A variance inflation factor test was performed to assess if multicollinearity was present in the model.

Logistic regression was used to study the relationship between the 4 intersectional groups and kidney damage. The baseline group was defined as the one with lower kidney damage prevalence. Multivariate logistic regression analysis was performed to adjust for the same covariates included in the linear regression model (except renal function) for its known association with kidney damage that could confound the association. The odds ratio (OR) was used as a measure of effect for the odds of kidney damage for each group with respect to the baseline. A likelihood test ratio was performed to further assess if the multivariate logistic regression model was improved, taking into account the intersection between sex and ethnicity. The Hosmer-Lemeshow test was used for analyzing goodness of fit.

To assess the robustness and consistency of our findings, the following sensitivity analyses were conducted: using different baseline categories in both regression models, including type of antihypertensive treatment instead of the generic treatment variable, using logarithmic values for those relevant variables that were skewed, and excluding individuals under medication that could interfere with the RAAS (renin-angiotensin-aldosterone system). We further addressed differences between the multivariate models performed with the same model but including the variable sex and ethnicity separately and addressing their interaction.

Moreover, to further adjust for the mentioned covariates included in the models, we performed an inverse probability weighting (IPW) analysis through a propensity score adjustment. IPW is a statistical technique that allows to further adjust for confounding, creating weights to balance confounders across the 4 intersectional groups, reducing bias and getting closer to causality. For this purpose, 4 propensity scores (one for each intersectional group) were calculated based on regressions including the same covariates from the previous models. Inverse weights (1/propensity score) were calculated to balance the distribution of confounding factors between the 4 intersectional categories. Given the intersectional nature of the exposure, our analyses aimed to capture subgroup-specific associations without requiring further stratification.

*P*<0.05 was considered statistically significant. Statistical analysis was performed using the Stata software (version 18.0; StataCorp LLC, College Station, TX).

## Results

The study included 654 patients with hypertension, with a mean age of 44 years (SD, 13.6), 250 (38.23%) women, and 404 (61.77%) of Black ethnicity. Among them, mean cf-PWV was 9.92 (2.33) m/s, and 22.78% had kidney damage.

After stratifying the population by sex and ethnicity, blood pressure was higher in Black men (152±19/93±14 mm Hg), followed by Black women (148±19/91±11 mm Hg), White men (146±16/88±11 mm Hg), and White women (142±17/90±13 mm Hg), *P*<0.01. Differences were also found between the 4 groups regarding lipid profile, HbA1c levels, body mass index, aldosterone, renin, hypertension treatment, and smoking status (Table 1).

**Table 1.**
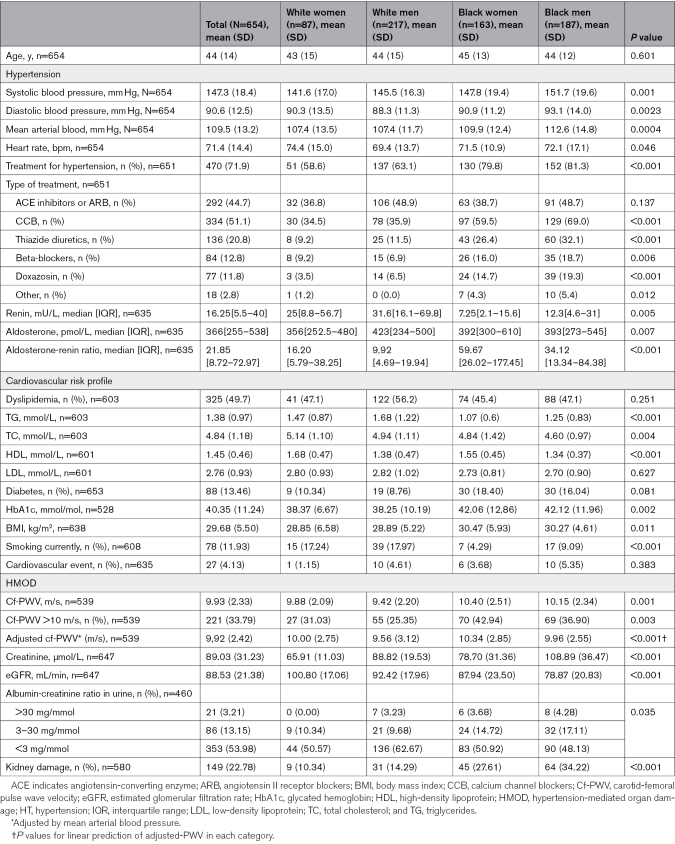
Characteristics of 654 Patients With Hypertension and Their Intersectional Differences

Cf-PWV was higher in Black women (10.4±2.5 m/s), followed by Black men (10.1±2.3 m/s), White women (9.9±2.1 m/s), and White men (9.4±2.2 m/s, *P*<0.01). Prevalence of kidney damage was higher in Black men (34.2%), followed by Black women (27.6%), White men (14.3%), and White women (10.3%, χ^2^ *P*<0.01; Table 1). Distribution of the prevalence of kidney damage by eGFR categories and albumin/creatinine ratio is shown in Table S1 and Figure S1.

### Aldosterone and HMOD

Aldosterone was higher in Black individuals compared with White individuals, with no significant differences between men and women (Table 1).

**Figure 1.**
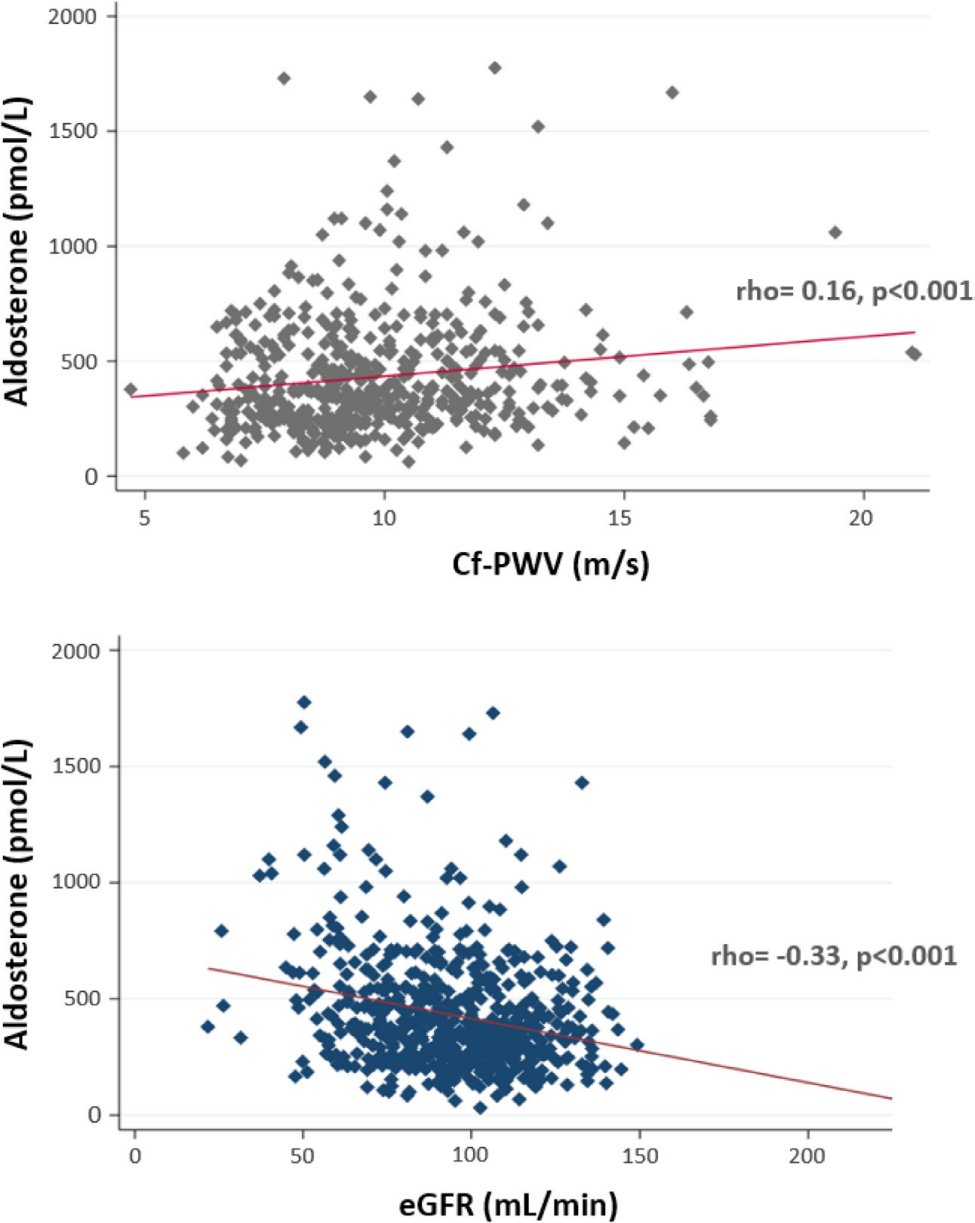
**Correlation between aldosterone, pulse wave velocity, and estimated glomerular filtration rate (eGFR) in the overall study population, adjusted by age and mean arterial blood pressure.** Cf-PWV indicates carotid-femoral pulse wave velocity.

**Figure 2.**
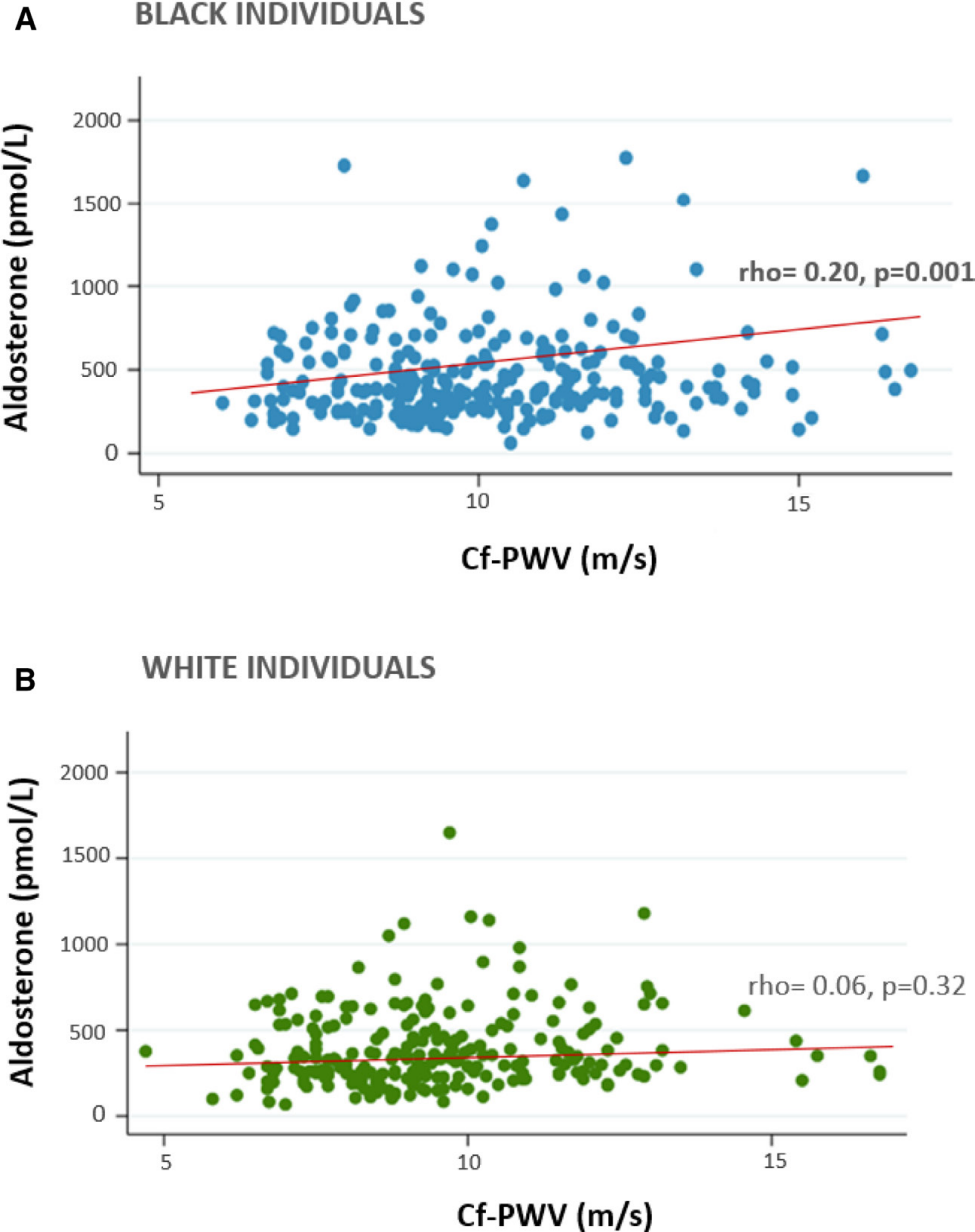
**Correlation between aldosterone and pulse wave velocity according to ethnicity. A**, Black individuals and (**B**) White individuals, adjusted by age and mean arterial blood pressure. Cf-PWV indicates carotid-femoral pulse wave velocity.

In the entire study population, there was a positive adjusted correlation between aldosterone level and cf-PWV (*ρ*=0.16, *P*<0.001), with stratified analysis by sex showing a similar correlation between men and women (Figure 1; Table 2). After stratifying the population according to ethnicity, the correlation between aldosterone and cf-PWV persisted only in Black individuals (*ρ*=20, *P*=0.001; Figure 2; Table 2; Table S2). The same results were obtained when correlating Black women and Black men separately, as well as White women and White men. In relation to kidney function, a negative correlation between aldosterone and eGFR was seen in the entire population (*ρ*=−0.33, *P*<0.001) and persisted after stratification for sex and ethnicity (Figure 1; Table 2).

**Table 2.**
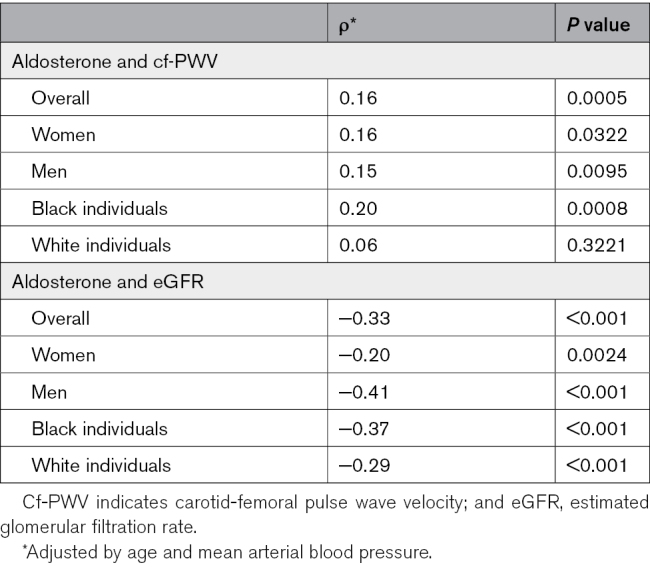
Pearson Correlations for the Association Between eGFR and PWV With Aldosterone, Adjusted by Sex and Ethnicity

### Multivariate Regression Analyses and IPW Results

Multivariate linear regression analysis adjusted for body mass index, mean arterial blood pressure, heart rate, diabetes, dyslipidemia, creatinine, pharmacological treatment, smoking, aldosterone, and renin showed that women of both ethnicities had higher cf-PWV (White β=0.7 m/s, *P*=0.030, Black β=0.5 m/s, *P*=0.046) compared with White men used as reference. Other variables that were found to be independently associated with cf-PWV in the final multivariate model were age, diabetes, blood pressure, heart rate, and renal function (Table 3). Variance inflation factor showed no strong correlation between the explanatory variables, and *F* test resulted in a good overall significance of the regression model with all the covariates included compared with a model without covariates (*P*<0.001). After performing the IPW analysis, only Black women maintained the significant association (β=1.25, *P*<0.001; Table S3). The use of intersectional categories in IPW allowed us to directly assess heterogeneity in PWV across sex and ethnic subgroups, which in this case only remained in Black women.

**Table 3.**
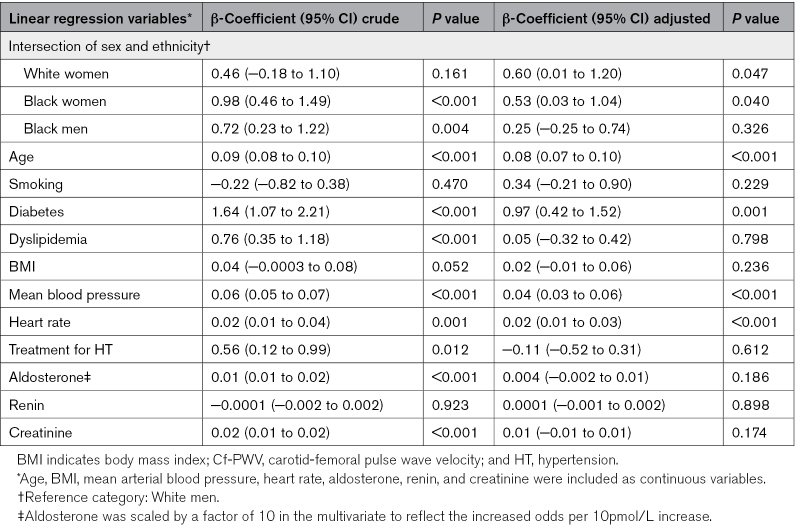
Final Multivariate Linear Regression Model, for the Intersection of Sex and Ethnicity and Its Association With cf-PWV

Multivariate logistic regression adjusted by the same covariates as in the previous regression except renal function, showed that the OR of kidney damage was higher in Black men at 3.96 ([95% CI, 1.50–10.45]; *P*=0.005), followed by Black women at 3.09 ([95% CI, 1.14–8.35]; *P*=0.026), compared with White women. Other variables that were found to be independently associated with kidney damage in the final multivariate model were age, mean blood pressure, and aldosterone (Table 4). For each 10 pmol/L increase in serum aldosterone level, there was a 2% increase in the odds of kidney damage (*P*<0.001). Hosmer-Lemeshow test resulted in a good fit of the model, and likelihood test ratio showed that the model was improved when all the covariates were included. When performing the IPW analysis, the results were similar (Black men OR, 4.16; *P*=0.007 and Black women OR, 3.83; *P*=0.009; Table S4).

**Table 4.**
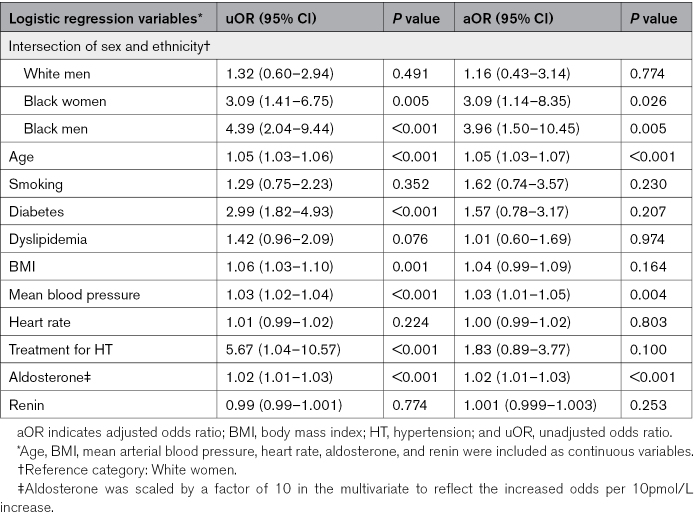
Final Multivariate Logistic Regression Model, for the Intersection of Sex and Ethnicity and Its Association With Kidney Damage

When changing the baseline categories in both regressions for sensitivity analysis, the main associations for the intersection of sex and ethnicity remained consistent for both multivariate regressions (Tables S5 and S6). Including the type of antihypertensives did not change the associations in both regression models (Tables S7 and S8). Multivariate models that included sex and ethnicity separately were consistent with the results from the multivariate model presented (Tables S9 and S10). Using logarithmic values for renin, and for renin and aldosterone, had consistent results and significant associations in both multivariate models. Exclusion of individuals who were under medications that interfere with RAAS (angiotensin-converting enzyme inhibitors and angiotensin II receptor blockers), kept the same significant associations including the one found with aldosterone in the logistic regression for kidney damage (*P*=0.006).

## Discussion

Our cross-sectional, intersectional analysis in a dual-ethnic cohort of individuals with primary hypertension reveals that the burden of renal and vascular HMOD varies by both ethnicity and sex.

Despite lower blood pressure values, women had higher values of cf-PWV compared with men (Table 1). After taking into account multiple confounders, and with further adjustments for weights with the IPW analysis, cf-PWV remained higher in Black women (increase in 1.25 m/s, *P*<0.001) compared with White men. The existing literature has shown that arterial stiffness increases after menopause^11,21^ and is associated with increased risk of developing cardiovascular events.^22^ However, data in the younger population with and without hypertension are conflicting.^23–25^ Similarly, it has been suggested that increased arterial stiffness could be more common in African-origin subjects, although this has often been linked to a cluster of cardiovascular risk and socioeconomic factors.^26–29^ Our cross-sectional analysis is in line with the existing literature and shows that arterial stiffness is higher in women, especially in Black women, after full adjustments. However, whether an elevated arterial stiffness in Black women might be implicated in the pathogenesis of cardiovascular disease in this group would need to be elucidated.^30,31^

Prevalence of kidney damage was higher in Black individuals compared with White individuals, irrespective of sex, and the odds persisted after adjustment for confounders and weights (OR of 3.83 for Black women and 4.16 for Black men; *P*<0.01 when compared with White women). Ethnic disparities in the development of renal impairment have been predominantly documented among Black populations,^32–34^ where longitudinal studies have shown that Black individuals have increased mortality rates in hypertension-related end-stage renal disease.^35^ However, it is important to recognize that ethnicity is a complex construct, heavily influenced by environmental and contextual factors. As such, data obtained from one geographic region may not be generalizable to other settings. Notably, other factors such as genetic polymorphisms associated with an increased risk of chronic kidney disease, *MYH9* (myosin heavy chain 9) and *APOL1* (apolipoprotein L1) genes, have been reported to occur more frequently in individuals of African ancestry.^36–38^ Our findings are consistent with these observations, in Black individuals in the United Kingdom, and showed no significant interaction between ethnicity and sex in relation to renal-HMOD.

Regarding aldosterone associations, our results do not unequivocally establish whether aldosterone is a significant independent factor associated with HMOD in relation to ethnic and sex differences. Regarding renal-HMOD, aldosterone appears to be independently associated with kidney damage, with this association being consistent across ethnic and sex groups. In relation to vascular HMOD, the modest correlation observed in Black individuals should be interpreted with caution, given the limitations of cross-sectional analysis.

Aside from its hemodynamic action, aldosterone is known to modulate cell proliferation, inflammation, and fibrosis,^39^ and it has been shown to be associated with chronic kidney disease in the general population, but little attention has been given in the literature on renal and vascular HMOD.^40^ In a previous study, we demonstrated that aldosterone was associated with adverse left ventricular remodeling in Black but not in White individuals.^9^ In the present study, we found that aldosterone was associated with kidney damage, regardless of ethnicity and sex, after adjustment for confounders, with a 10% increase in the odds of kidney damage for every 50 pmol/L increase in aldosterone (Table 4). These findings, although speculative given the cross-sectional design, are in line with observations by Verma et al, who reported an association between aldosterone levels and kidney disease progression in a large cohort study.^40^

The association of aldosterone and vascular stiffness has been less investigated,^41,42^ and there are no specific human data on ethnic differences. Aldosterone might be implicated in vascular dysfunction and remodeling through immune activation, inflammation, and oxidative stress, mechanisms that may promote arterial stiffness.^43,44^ Whether such pathways could, in theory, vary between ethnicities due to differences in renin–aldosterone system physiology, warrants further investigation.

The study had several limitations that should be mentioned. First, the cross-sectional design could not support a definite conclusion regarding the causality of the findings. Second, in this cohort, there were no variables related to socioeconomic status, lifestyle factors, and additional confounders, such as duration of antihypertensive treatment, diet, and physical activity. Third, the aldosterone level was measured only at a single time point at baseline, and not during a period of controlled sodium intake, with some individuals under treatment affecting the RAAS. Although the sensitivity analyses were consistent with the findings in the whole study population, we cannot exclude that these factors may have influenced aldosterone-related findings. Fourth, adjusted-aldosterone correlations with HMOD and by ethnicity are modest and do not support conclusive evidence on causality nor effect modification. Finally, a selection bias may have occurred based on our enrollment strategy.

However, this study also had multiple strengths, including the incorporation of a comprehensive set of clinical variables derived from a relatively large clinical cohort of patients and the introduction of a novel intersectional approach to assessing sex and ethnic differences in HMOD.

### Conclusions

There are ethnic and sex differences in renal and vascular HMOD, with Black individuals presenting increased odds of kidney impairment and Black women showing higher arterial stiffness. Whether aldosterone might be a contributing factor for the ethnic and sex differences in HMOD would require further investigations.

### Perspectives

The findings reveal that ethnic and sex differences are relevant in HMOD at renal and vascular levels. This is a step forward in understanding the pathophysiology of HMOD, which could help the future development of precision and personalized medicine in hypertension.

## ARTICLE INFORMATION

### Acknowledgments

The authors sincerely thank all the participants for their involvement and contributions to this study. The authors also want to acknowledge the Clinical Pharmacology Unit at the School of Cardiovascular and Metabolic Medicine & Sciences, King’s College London, for their valuable support in participant recruitment, data collection, and management. Lastly, the authors acknowledge the Spanish Internal Medicine Society and Foundation for their partial funding of Dr Hernández-Rubio’s postdoctoral placement at the School of Cardiovascular and Metabolic Medicine & Sciences, King’s College London.

### Sources of Funding

This study was supported by a stratified medicines grant from the Medical Research Council: MR/M016560/1.

### Disclosures

None.

### Supplemental Material

Tables S1–S10

Figure S1

STROBE Checklist

## Supplementary Material


