# Supplementary material for: Role of Ethnicity and Sex in Hypertension-Mediated Organ Damage in a Dual-Ethnic Cohort of Individuals With Hypertension
Source: Hypertension. 2025 Oct 20;83(2):e25032. doi: 10.1161/HYPERTENSIONAHA.125.25032 (PMC12822773; doi:10.1161/HYPERTENSIONAHA.125.25032)
Supplement: Supplementary file 1 [file hyp-83-e25032-s001.docx]

**"The role of Ethnicity and Sex in hypertension-mediated organ damage in a dual-ethnic cohort of Hypertensive individuals"**

**SUPPLEMENTAL MATERIAL**

**Authors:**

Anna Hernandez-Rubio, MD, PhD ^1, 2^

Ryan McNally, PhD^3^

Núria Pedrós Barnils, PhD^4^

Bushra Farukh^1^

Phil Chowienczyk ^1^ , Full Professor, MD, PhD

J Kennedy Cruickshank^1^, Full Professor, MD, PhD

Luca Faconti^1*^, MD, PhD

^1^School of Cardiovascular and Metabolic Medicine & Sciences, King's College London British Heart Foundation Centre, London, UK.

^2^Germans Trias i Pujol Research Institute, Badalona, Barcelona, Spain.

^3^King’s Health Partners, Centre for Translational Medicine, King’s College London, London, UK.

^4^Institute for Public Health and Nursing Research, University of Bremen, Germany.

***Correspondence**

Dr. Luca Faconti, MD, PhD.

[luca.faconti@kcl.ac.uk](mailto:luca.faconti@kcl.ac.uk)

Clinical Lecturer in Clinical Pharmacology, King's College London

School of cardiovascular and metabolic medicine & sciences

Consultant (Honorary) in Hypertension, Guy's and St Thomas' NHS Foundation Trust

St. Thomas Hospital, 4th Floor, North Wing

Westminster Bridge Road, London, SE1 7EH

Ext: 84768

# SUPPLEMENTAL TABLES

**Table S1.** Number of hypertensive individuals included in each kidney damage subcategory depending on estimated glomerular filtration rate and albumin-creatinine ratio.

| **eGFR categories (mL/min)** | **ACR (mg/mmol)** | | |
| --- | --- | --- | --- |
|  | **<3** | **3-30** | **>30** |
| **>=90 G1** | 215 | 36 | 8 |
| **60-89 G2** | 117 | 35 | 9 |
| **45-59 G3a** | 15 | 11 | 3 |
| **30-44 G3b** | 1 | 2 | 1 |
| **15-29 G4** | 1 | 2 | 0 |
| **<15 G5** | 0 | 0 | 0 |

Abbreviations: ACR, albumin-creatinine ratio in urine; eGFR, estimated glomerular filtration rate.

Note: Kidney damage categories are defined according to the 2024 KDIGO guidelines including both eGFR and ACR as follows: G1 (normal/high renal function) for eGFR >90 mL/min/1.73 m², G2 (mildly decreased) for 60–90 mL/min/1.73 m², G3 (moderately decreased) for 30–60 mL/min/1.73 m², and G4/G5 (severely decreased function or kidney failure) for <30 mL/min/1.73 m². For each eGFR category, additional categorisation has been made for ACR following the three groups shown in the Table.

**Table S2.** Association between arterial stiffness (>10m/s of carotid-femoral pulse-wave velocity) and aldosterone levels (highest vs lowest tertile) by ethnic group.

| **Crude** | | **Black individuals** | | **White individuals** | |
| --- | --- | --- | --- | --- | --- |
| OR (95% CI) | p-value | OR (95% CI) | p-value | OR (95% CI) | p-value |
| **2.07** (1.35-3.19) | 0.001 | **2.24** (1.23-4.08) | 0.008 | **1.59** (0.83-3.03) | 0.16 |

Abbreviations: OR, Odds Ratio; CI, Confidence Interval.

**Table S3.** Inverse probability weighting analysis for the association between the intersection of sex and ethnicity and cf-PWV.

|  | **β-Coefficient (95% CI)** | **p-value** |
| --- | --- | --- |
| **Intersection of sex and ethnicity *** | | |
| White women | 0.15(-0.59, 0.89) | 0.688 |
| Black women | **1.25(0.77, 1.73)** | **<0.001** |
| Black men | 0.25(-0.25, 0.74) | 0.326 |

*Reference category White men

Abbreviations: CI, Confidence Interval.

**Table S4.** Inverse probability weighting analysis for the association between the intersection of sex and ethnicity and kidney damage.

|  | **OR (95% CI)** | **p-value** |
| --- | --- | --- |
| **Intersection of sex and ethnicity *** | | |
| White men | 0.59(0.12-3.01) | 0.527 |
| Black women | **3.83(1.40-10.48)** | **0.009** |
| Black men | **4.16(1.48-11.69)** | **0.007** |

*Reference category White women

Abbreviations: OR, Odds Ratio; CI, Confidence Interval.

**Table S5.** Association between the intersectional categories and cf-PWV with different reference groups.

| **Multivariate linear Regression for cf-PWV** | **β-Coefficient (95% CI)** | **p-value** |
| --- | --- | --- |
| **Intersection of sex and ethnicity: White men baseline** | | |
| White women | 0.60(0.01, 1.20) | 0.047 |
| Black women | 0.53(0.03, 1.04) | 0.040 |
| Black men | 0.25(-0.25, 0.74) | 0.326 |
| **Intersection of sex and ethnicity: White women baseline** | | |
| White men | -0.60(-1.20, -0.01) | 0.047 |
| Black women | -0.07(-0.69, 0.55) | 0.825 |
| Black men | -0.35(-1.04, 0.33) | 0.307 |
| **Intersection of sex and ethnicity: Black men baseline** | | |
| White women | 0.35(-0.32, 1.04) | 0.307 |
| White men | -0.25(-0.74, 0.25) | 0.326 |
| Black women | 0.28(-0.26, 0.83) | 0.309 |
| **Intersection of sex and ethnicity: Black women baseline** | | |
| White women | 0.07(-0.55, 0.69) | 0.825 |
| White men | -0.53(-1.04, -0.03) | 0.040 |
| Black men | -0.28(-0.83, 0.26) | 0.309 |

Abbreviations: CI, Confidence Interval; cf-PWV, carotid-femoral Pulse Wave Velocity.

**Table S6.** Association between the intersectional categories and kidney damage with different reference groups.

| **Multivariate logistic Regression for Kidney damage** | **OR (95% CI)** | **p-value** |
| --- | --- | --- |
| **Intersection of sex and ethnicity: White men baseline** | | |
| White women | 0.86(0.32-2.34) | 0.774 |
| Black women | 2.67(1.31-5.45) | 0.007 |
| Black men | 3.42(1.74-6.73) | <0.001 |
| **Intersection of sex and ethnicity: White women baseline** | | |
| White men | 1.16(0.43-3.14) | 0.774 |
| Black women | 3.09(1.14-8.35) | 0.026 |
| Black men | 3.96(1.50-10.45) | 0.005 |
| **Intersection of sex and ethnicity: Black men baseline** | | |
| White women | 0.25(0.10-0.67) | 0.005 |
| White men | 0.29(0.15-0.58) | <0.001 |
| Black women | 0.78(0.42-1.44) | 0.426 |
| **Intersection of sex and ethnicity: Black women baseline** | | |
| White women | 0.32(0.12-0.88) | 0.026 |
| White men | 0.37(0.18-0.77) | 0.007 |
| Black men | 1.28(0.69-2.36) | 0.426 |

Abbreviations: OR, Odds Ratio; CI, Confidence Interval.

**Table S7.** Multivariate linear regression model for the association between ethnicity and sex with Cf-PWV, including type of antihypertensive treatment.

| **Linear Regression variables** | **β-Coefficient (95% CI)** | **p-value** |
| --- | --- | --- |
| **Intersection of sex and ethnicity *** | | |
| White women | **0.62(0.01, 1.22)** | **0.047** |
| Black women | **0.53(0.01, 1.06)** | **0.048** |
| Black men | 0.25(-0.27, 0.77) | 0.343 |
| **Age** | **0.08(0.07, 0.10)** | **<0.001** |
| **Smoking** | 0.37(-0.20, 0.93) | 0.202 |
| **Diabetes** | **0.99(0.43, 1.56)** | **0.001** |
| **Dyslipidemia** | 0.05(-0.32, 0.43) | 0.785 |
| **BMI** | 0.02(-0.02, 0.06) | 0.253 |
| **Mean Blood Pressure** | **0.04(0.03, 0.06)** | **<0.001** |
| **Heart Rate** | **0.02(0.01, 0.03)** | **<0.001** |
| **Aldosterone** | 0.0004(-0.0002, 0.001) | 0.246 |
| **Renin** | 0.0001(-0.001, 0.002) | 0.998 |
| **Creatinine** | 0.01(-0.01, 0.01) | 0.220 |
| **Hypertensive treatment:** | | |
| **ACEI or ARB** | 0.006(-0.39, 0.43) | 0.975 |
| **CCB** | -0.03(-0.41, 0.36) | 0.887 |
| **Thiazide diuretics** | -0.13(-0.65, 0.39) | 0.627 |
| **Beta-blockers** | -0.19(-0.81, 0.42) | 0.534 |
| **Doxazosine** | 0.22(-0.47, 0.91) | 0.528 |
| **Other antihypertensive** | 0.20(-1.06, 1.46) | 0.758 |

*Reference category White men

Abbreviations: ACEI, Angiotensin-Converting Enzyme Inhibitors; ARB, Angiotensin II Receptor Blockers; BMI, Body Mass Index; CCB, Calcium Channel Blockers, CI, Confidence Interval; Cf-PWV, Carotid-femoral Pulse Wave Velocity.

**Table S8.** Multivariate logistic regression model for the association between ethnicity and sex with kidney damage, including type of antihypertensive treatment.

| **Logistic Regression variables** | **aOR (95% CI)** | **p-value** |
| --- | --- | --- |
| **Intersection of sex and ethnicity *** | | |
| White men | 1.26(0.46-3.43) | 0.650 |
| Black women | **2.78(1.03-7.55)** | **0.044** |
| Black men | **3.38(1.27-9.04)** | **0.015** |
| **Age** | **1.04(1.01-1.05)** | **<0.001** |
| **Smoking** | 1.38(0.61-3.11) | 0.442 |
| **Diabetes** | 1.28(0.60-2.71) | 0.522 |
| **Dyslipidemia** | 1.01(0.59-1.73) | 0.985 |
| **BMI** | 1.03(0.98-1.08) | 0.294 |
| **Mean Blood Pressure** | **1.02(1.01-1.05)** | **0.024** |
| **Heart Rate** | 1.01(0.99-1.02) | 0.262 |
| **Aldosterone** | **1.002(1.001-1.003)** | **<0.001** |
| **Renin** | 1.002(0.999-1.004) | 0.189 |
| **Hypertensive treatment:** | | |
| **ACEI or ARB** | 1.2(0.67-2.16) | 0.539 |
| **CCB** | 1.43(0.82-2.48) | 0.209 |
| **Thiazide diuretics** | 0.71(0.36-1.42) | 0.331 |
| **Beta-blockers** | 2.56(1.68-7.52) | 0.001 |
| **Doxazosine** | 2.08(0.93-2.67) | 0.076 |
| **Other antihypertensive** | 6.77(0.67-8.43) | 0.105 |

*Reference category White women

Abbreviations: aOR, adjusted-Odds Ratio; ACEI, Angiotensin-Converting Enzyme Inhibitors; ARB, Angiotensin II Receptor Blockers; BMI, Body Mass Index; CCB, Calcium Channel Blockers, CI, Confidence Interval.

**Table S9**. Multivariate linear regression model with the interaction term between sex and ethnicity on its association with cf-PWV.

| **Linear Regression variables** | **β-Coefficient (95% CI)** | **p-value** |
| --- | --- | --- |
| **Ethnicity (black)** | 0.25(-0.25, 0.74) | 0.406 |
| **Sex (women)** | 0.60(0.01, 1.20) | 0.047 |
| **Ethnicity(black) # Sex(women)** | -0.32(-1.07, 0.43) | 0,406 |
| **Age** | 0.08(0.08, 0.10) | <0.001 |
| **Smoking** | 0.34(-0.21, 0.89) | 0.229 |
| **Diabetes** | 0.97(0.42, 1.52) | 0.001 |
| **Dyslipidemia** | 0.05(-0.32, 0.42) | 0.798 |
| **BMI** | 0.02(-0.01, 0.06) | 0.236 |
| **Mean Blood Pressure** | 0.04(0.01, 0.03) | <0.001 |
| **Heart Rate** | 0.02(0.01, 0.03) | <0.001 |
| **Treatment for HT** | 0.11(-0.52, 0.31) | 0.612 |
| **Aldosterone** | 0.004(0.002, 0.009) | <0.186 |
| **Renin** | 0.0001(-0.001, 0.002) | 0.898 |
| **Creatinine** | 0.01(0.002, 0.001) | 0.174 |

Abbreviations: CI, Confidence Interval; HT, Hypertension; BMI, Body Mass Index.

**Table S10.** Multivariate logistic regression model with the interaction term between sex and ethnicity on its association with kidney damage.

| **Logistic Regression variables** | **aOR (95% CI)** | **p-value** |
| --- | --- | --- |
| **Ethnicity (black)** | **3.42(1.74-6.73)** | **<0.001** |
| **Sex (women)** | 0.86(0.32-2.34) | 0.774 |
| **Ethnicity(black) # Sex(women)** | 0.90(0.28-2.91) | 0.864 |
| **Age** | 1.05(1.03-1.07) | <0.001 |
| **Smoking** | 1.62(0.74-3.57) | 0.230 |
| **Diabetes** | 1.57(0.78-3.17) | 0.207 |
| **Dyslipidemia** | 1.01(0.60-1.69) | 0.974 |
| **BMI** | 1.04(0.99-1.09) | 0.164 |
| **Mean Blood Pressure** | 1.03(1.01-1.05) | 0.004 |
| **Heart Rate** | 1.00(0.99-1.02) | 0.803 |
| **Treatment for HT** | 1.83(0.89-3.77) | 0.100 |
| **Aldosterone** | 1.002(1.001-1.003) | <0.001 |
| **Renin** | 1.001(0.999-1.003) | 0.253 |

Abbreviations: OR, Odds Ratio; CI, Confidence Interval; HT, Hypertension; BMI, Body Mass Index.

# SUPPLEMENTAL FIGURES

**Figure S1.** Distribution of participants across estimated glomerular filtration rate (eGFR) categories, stratified by intersectional categories.


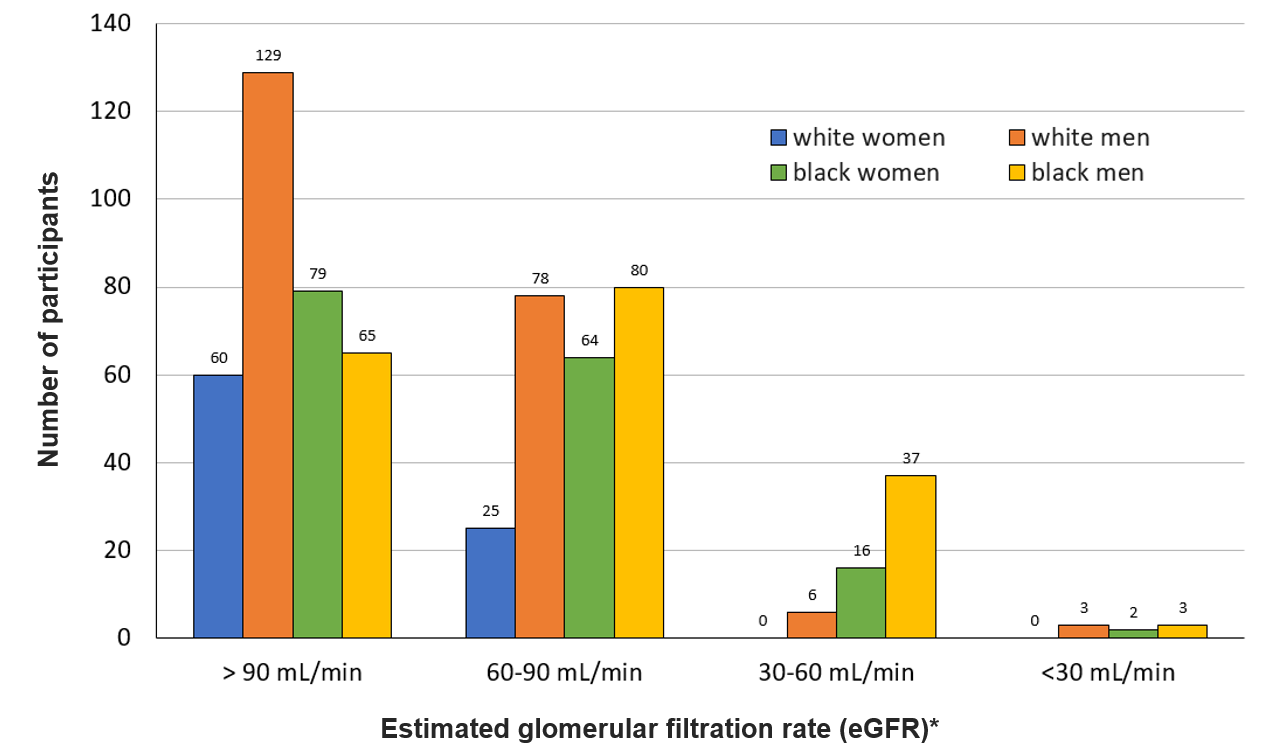


Abbreviations: eGFR, estimated glomerular filtration rate.

*Kidney function categories are defined according to the 2024 KDIGO guidelines as follows: G1 (normal/high renal function) for eGFR >90 mL/min/1.73 m², G2 (mildly decreased) for 60–90 mL/min/1.73 m², G3 (moderately decreased) for 30–60 mL/min/1.73 m², and G4/G5 (severely decreased function or kidney failure) for <30 mL/min/1.73 m².
